# Universal Human Papillomavirus Typing Assay: Whole-Genome Sequencing following Target Enrichment

**DOI:** 10.1128/JCM.02132-16

**Published:** 2017-02-22

**Authors:** Tengguo Li, Elizabeth R. Unger, Dhwani Batra, Mili Sheth, Martin Steinau, Jean Jasinski, Jennifer Jones, Mangalathu S. Rajeevan

**Affiliations:** a Division of High-Consequence Pathogens & Pathology, Centers for Disease Control and Prevention, Atlanta, Georgia, USA; b Division of Scientific Resources, Centers for Disease Control and Prevention, Atlanta, Georgia, USA; c Agilent Technologies, Inc., Santa Clara, California, USA; Memorial Sloan-Kettering Cancer Center

**Keywords:** HPV typing, broad-spectrum assay, whole-genome sequencing, target enrichment

## Abstract

We designed a universal human papillomavirus (HPV) typing assay based on target enrichment and whole-genome sequencing (eWGS). The RNA bait included 23,941 probes targeting 191 HPV types and 12 probes targeting beta-globin as a control. We used the Agilent SureSelect XT2 protocol for library preparation, Illumina HiSeq 2500 for sequencing, and CLC Genomics Workbench for sequence analysis. Mapping stringency for type assignment was determined based on 8 (6 HPV-positive and 2 HPV-negative) control samples. Using the optimal mapping conditions, types were assigned to 24 blinded samples. eWGS results were 100% concordant with Linear Array (LA) genotyping results for 9 plasmid samples and fully or partially concordant for 9 of the 15 cervical-vaginal samples, with 95.83% overall type-specific concordance for LA genotyping. eWGS identified 7 HPV types not included in the LA genotyping. Since this method does not involve degenerate primers targeting HPV genomic regions, PCR bias in genotype detection is minimized. With further refinements aimed at reducing cost and increasing throughput, this first application of eWGS for universal HPV typing could be a useful method to elucidate HPV epidemiology.

## INTRODUCTION

Human papillomaviruses (HPV) are double-stranded DNA viruses in the family Papillomaviridae. More than 200 genotypes are recognized based on the sequence of the approximately 8-kbp circular genome, with variants in each genotype based on sequence relatedness (1, 2). Detection and typing of HPV has clinical and public health significance because of the association of some genotypes in the Alphapapillomavirus genus with anogenital and oropharyngeal cancers. As a result, most assays used in HPV epidemiology and natural history studies are directed to these genotypes, and many PCR-based assays use degenerate primers directed to the L1 region of the HPV genome (3, 4). Studies examining disease associations of an increasingly broad spectrum of HPV genotypes have been hampered by the need for multiple assays to detect genotypes in alpha, beta, and gamma genera (5, 6).

Next-generation sequencing (NGS)-based methods have the promise to improve the spectrum of HPV genotypes detected. Some NGS methods used PCR with consensus primers targeting the L1 region for library preparation, restricting regions of the genome that could be analyzed (7–13). HPV genome sequencing to single or limited HPV genotypes was achieved using the standard protocol for library preparation without any target enrichment (14) or after whole-genome amplification by rolling-circle amplification (15) or highly multiplexed degenerate primers (16). There are a few reports of NGS using target enrichment to study the mechanistic signatures of integration of a limited number of HPV genotypes in cervical carcinomas (17–19). The goal of this study was to capitalize on the availability of new target enrichment technology to facilitate whole-genome sequencing to detect, genotype, and potentially characterize variant and integration statuses of all known HPVs belonging to alpha, beta, and gamma genera using a single assay. Target enrichment technology uses hybridization to purify genomic fragments of interest with DNA or RNA baits. DNA/RNA baits were initially used and proved to be highly effective to enrich human exomes for variant calling by deep sequencing (20, 21). We developed custom RNA baits specific to all known HPV genomes to enrich the sample for sequencing of whole viral genomes. Here, we describe and provide an initial evaluation of this whole-genome sequence-based approach for broad-spectrum HPV genotype determination in samples with single and multiple HPV infection using the Agilent SureSelect target enrichment technology.

## RESULTS

### Assessment of sequence data quality.

**FIG 1:**
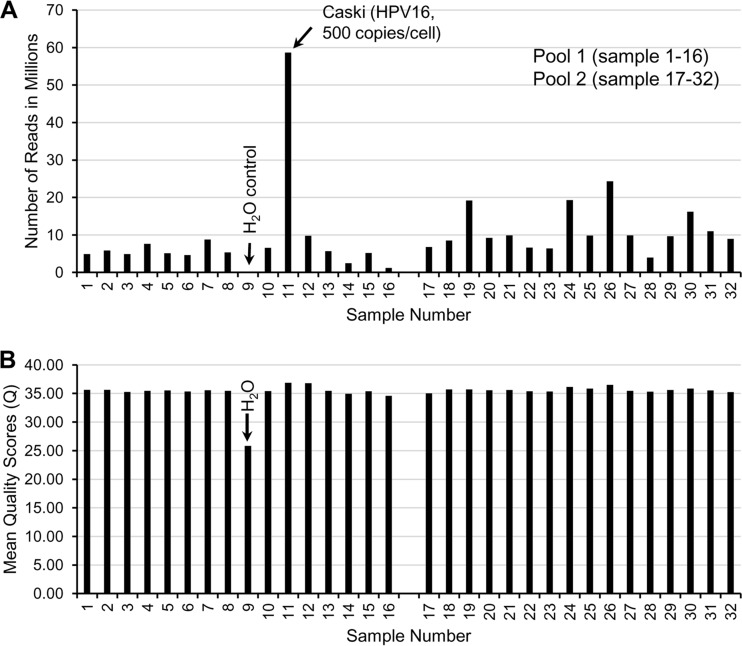
Number of reads (A) and mean base quality (Q) score of reads (B) passing the default filtering of Illumina BCl12fastq V1.8.4 software. Reads were restricted to 0 mismatches in 8-bp index reads.

DNA sheared to approximately 150 bp is expected to increase in size to around 300 bp in the indexed library after ligation of adaptors that enable limited PCR amplification and sequencing of the library. As expected, bioanalyzer analysis of the indexed pooled libraries prepared for sequencing indicated the size distribution with a peak around 300 bp. The seed concentration of 5.3 pM generated cluster densities of 1,173,000 and 1,183,000/mm^2^ in pool 1 (samples 1 to 16) and 2 (samples 17 to 32), respectively. All raw reads from both pools passed the default filtering of the Illumina BCl2fastq V1.8.4 software (pool 1, 268,096,524; pool 2, 226,336,668). The average number of reads per sample in pool 1 was 8,538,564, and that from Caski at 100 ng (sample 11) with ∼500 HPV16 copies/cell dominated, comprising 21.9% of the total (Fig. 1A). The average number of reads per sample in pool 2 was 11,224,892. The mean base quality (Q) score for each sample (excluding sample 9, the water control) ranged from 34.57 to 36.87 (mean Q of 35.6), and 88% of the bases had quality scores greater than 30 (Fig. 1B). The water control generated 3,692 reads with Q scores ranging from 2 to 40 (mean Q of 25), with only 47% of bases having a Q score greater than 30.

### Mapping results for internal control HBG.

**FIG 2:**
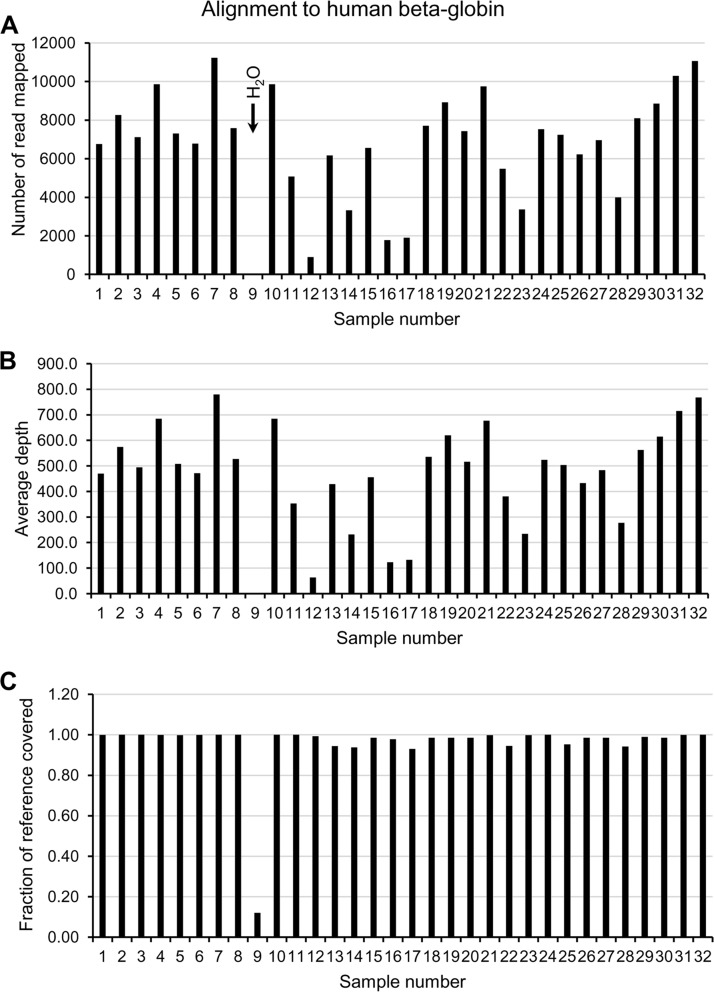
Performance of RNA baits for internal control human beta-globin gene based on number of reads (A), average coverage (B), and fraction of reference sequence covered by the reads (C).

The fraction of the globin reference sequence mapped in all samples with genomic DNA (all except the water control, sample 9) ranged from 93 to 100% (mean, 98%). Samples with 100 ng genomic DNA (samples 1 to 8, 10 to 11, 13, 15, and 17 to 32) generated a mean of 7,406 reads (range, 1,903 to 11,225) that, using L1-S1 stringency, mapped to the globin reference (human beta-globin [HBG] nucleotides [nt] 2041 to 3480) with an average depth of coverage ranging from 132.2 to 779.5 (mean coverage, 514.3) (Fig. 2). The number of mapped reads (range, 902 to 3,327; mean, 2,000.3) and coverage (range, 62.6 to 231; mean, 138.9) were reduced in samples with 10 ng genomic DNA (samples 12, 14, and 16). Only 2 reads from the water control (sample 9) mapped to beta-globin (depth of coverage of 0.1 and fraction of reference sequence mapped of 12%).

### Evaluating cutoff to improve signal/noise ratio for HPV genotyping from whole-genome sequence data.

**FIG 3:**
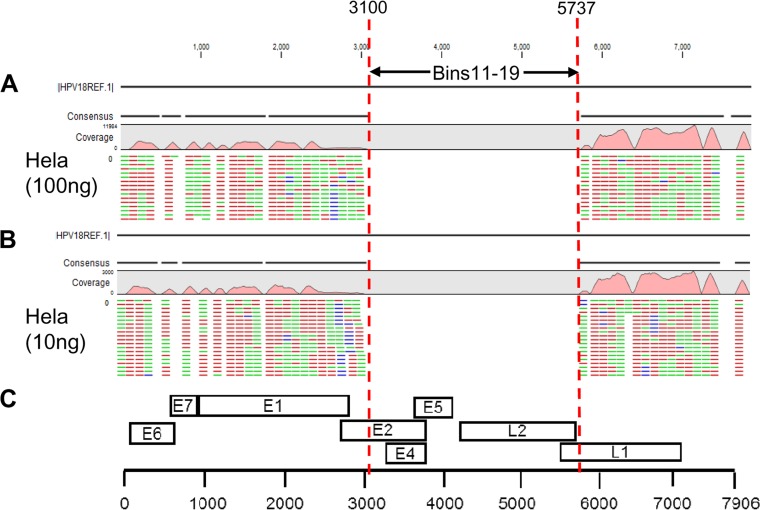
Mapping results showing high specificity of RNA baits restricted to the HPV18 genome integrated into the HeLa genome. The reduced fraction of HPV genome, covered by the sequenced reads due to deletion of the central region of the HPV18 genome (the central region indicated within the two dashed vertical lines), compatible with a deletion due to integration, is shown reproducibly with 100 ng (A) and 10 ng (B) input DNA. The stringent L1-S1 mapping conditions result in small gaps in the consensus sequence due to mismatched reads. Mapping results schematically aligned to HPV genome with the location of early and late region genes (C).

We evaluated mapping results for the 8 control samples (Table 1) in terms of the number of reads, average coverage, and the fraction of genome covered using different stringencies to differentiate signal from noise for determination of HPV genotype. Caski (100 ng) generated a total of 50,890,604 reads mapped to any HPV reference sequence, of which 99.94% of reads (50,861,070 reads) mapped specifically to HPV16 under the less stringent L0.5-S0.8 mapping condition. Whole-genome sequencing (WGS) also detected HPV16 in SiHa (134,664 out of 164,138 total reads; 82.04%) cells and HPV18 in HeLa (314,969 out of 342,887 total reads; 91.86%) cells as the most dominant types. Without any cutoffs for nonspecific signal, all positive controls detected additional HPV types under all three mapping stringencies. The presence of nonspecific signal was also seen in water and placental DNA negative controls. For example, WGS detected HPV16 with 1,800 reads in the negative placental DNA using L0.5-S0.8. With increased mapping stringency (L1-S1), placental DNA still detected HPV16 with 861 reads and average coverage of 10.9. Based on this, we selected a cutoff of ≥1,000 mapped reads and average coverage of ≥20 for reliable sequence assignment. We also added the fraction of reference genome covered of ≥0.5 (to indicate that at least 50% of the viral genome is retained, allowing for loss due to potential integration events) to the cutoff parameters. With L1-S1 mapping stringency and the selected cutoffs (number of mapped reads of ≥1,000, average coverage of ≥20, and fraction of genome covered of ≥0.5), HPV genotyping results for the 8 controls were concordant with the expected results (Table 1). The control cell line samples, Caski (∼500 HPV16 copies/cell), HeLa (∼50 HPV 18 copies/cell), and SiHa (1 to 2 HPV16 copies/cell), vary in known copy numbers of HPV and were analyzed at two concentrations of input DNA (100 ng and 10 ng). In each case, the number of mapped reads under stringent conditions roughly correlated with copy number (Table 1), and type assignment could be made with input of 10 ng DNA. The fraction of genome covered for HPV18 in the HeLa cell line was only 63%. No reads mapped to a 2.6-kbp central region (nt 3100 to 5730), compatible with a deletion (Fig. 3).

**TABLE 1:** Determination of cutoff to differentiate signal from noise in HPV whole-genome sequence data based on control samples

Control sample	Mapping results for stringency condition^*a*^:									HPV type	
	L0.5-S0.8			L0.5-S1			L1-S1				
	No. of reads mapped	Avg coverage	Fraction of genome covered	No. of reads mapped	Avg coverage	Fraction of genome covered	No. of reads mapped	Avg coverage	Fraction of genome covered	Without cutoff	With cutoff^*b*^
Caski (100 ng)	50,861,070	637,921.0	1.00	49,687,563	624,209.5	1.00	31,807,177	402,316.9	0.99	16	16
	187	2.1	0.63	139	1.7	0.57	116	1.5	0.50	33	
Caski (10 ng)	9,020,079	113,103.0	1.00	8,807,582	110,605.2	1.00	5,582,647	70,612.8	0.99	16	16
	37	0.4	0.16	14	0.2	0.12	10	0.1	0.10	45	
SiHa (100 ng)	134,664	1,679.0	1.00	130,955	1,638.0	1.00	87,797	1,110.5	0.98	16	16
	75	0.9	0.22	65	0.8	0.19	47	0.6	0.12	18	
SiHa (10 ng)	68,146	850.0	1.00	66,413	831.1	1.00	44,921	568.2	0.98	16	16
	94	1.0	0.33	66	0.8	0.30	59	0.7	0.29	31	
HeLa (100 ng)	314,969	3,953.7	0.67	298,994	3,760.7	0.67	183,032	2,329.5	0.64	18	18
	1,339	16.0	1.00	1,100	13.8	1.00	659	8.3	0.90	16	
HeLa (10 ng)	83,489	1,049.0	0.66	78,847	992.9	0.66	48,305	614.8	0.62	18	18
	610	7.3	0.99	409	5.1	0.98	225	2.8	0.77	16	
H_2_O	115	1.5	0.29	110	1.4	0.27	69	0.9	0.18	18	Negative
	12	0.1	0.13	2	0.0	0.02	1	0.0	0.01	16	
Placenta	1,800	21.3	1.00	1378	17.3	1.00	861	10.9	0.88	16	Negative
	46	0.5	0.26	37	0.5	0.24	23	0.3	0.16	18	

a Three mapping stringencies (L0.5-S0.8, L0.5-S1, and L1-S1) were evaluated using combination of parameters L and S, which represent read length (0.5 or 1) and similarity score (0.8 or 1), respectively.

b HPV types were determined following cutoff on number of mapped reads of ≥1,000, average coverage of ≥20, and fraction of genome covered of ≥0.5 under L1-S1 mapping stringency.

### Determination of HPV types from WGS data.

Enriched whole-genome sequencing (eWGS) data from the remaining 24 samples were analyzed using criteria set in the control samples to assign WGS HPV results without knowledge of prior HPV data. Comparing eWGS typing results with prior Linear Array (LA) results, type-specific HPV detection was at least partially concordant in 18 of 24 samples (75%) (Table 2). Genotype determinations for the 9 HPV plasmids (HPV45, -58, -31, -33, -52, -6, -18, -11, and -16) were 100% concordant. The average depth of coverage for the 9 WHO plasmids ranged from 1,210 to 3,751 with a mean of 2,837. Among the 15 cervicovaginal swab samples, typing results for 9 samples were fully or partially concordant. Of these 9, full concordance was found in 5 samples, 2 samples negative for HPV and 3 samples with multiple types (3, 4, and 8 types). Among the 6 discordant samples, 4 were negative by eWGS but positive for multiple types by LA, whereas 2 samples were HPV positive by both methods but for different types. The overall type-specific concordance for types included in the LA assay was 95.83%, and for LA-targeted types, there were no instances of samples positive by eWGS and negative by LA. In six samples, eWGS identified HPV types not included in the LA assay (HPV30, -43, -68a, -87, -90, -91, and -114). In cervicovaginal samples with multiple HPV types, the average depth of coverage varied greatly from as low as 27 to 129,998 (Table 2), probably a result of varying viral loads.

**TABLE 2:** HPV genotype determination in blinded samples based on target enrichment and whole-genome sequencing

Sample no.^*a*^	Sample type	No. of reads mapped	Avg coverage	Fraction of genome covered (≥0.5)	HPV type^*b*^		Concordance^*c*^
					WGS result	LA result	
1	Plasmid	282,773	3,598.5	0.99	**45**	**45**	Yes
2	Plasmid	293,528	3,751.6	1.00	**58**	**58**	Yes
3	Plasmid	210,381	2,659.0	1.00	**31**	**31**	Yes
4	Plasmid	190,903	2,413.7	0.99	**33**	**33**	Yes
5	Plasmid	237,806	2,994.3	1.00	**52**	**52**	Yes
6	Plasmid	268,460	3,357.4	1.00	**6**	**6**	Yes
7	Plasmid	95,100	1,210.4	1.00	**18**	**18**	Yes
8	Plasmid	169,355	2,135.4	1.00	**11**	**11**	Yes
17^*d*^	Cervicovaginal	16,120	208.7	0.16	34		Yes, partial
		1,736	379	0.85	**64 (34 subtype)**	**64 (reclassified as 34 subtype)**	
						81	
18	Cervicovaginal	7,873	100.9	0.99	**67**	**67**	Yes
		10,820	139.5	0.97	**54**	**54**	
		8,012	101.4	0.91	**70**	**70**	
		3,967	49.4	0.99	90*		
19	Cervicovaginal	667,344	8,554.6	0.90	**67**	**67**	Yes
		675,774	8,535.7	0.97	**42**	**42**	
		680,615	8,425.5	0.91	**89**	**89**	
		345,249	4,372.5	0.82	**59**	**59**	
		170,609	2,105.2	0.53	**83**	**83**	
		36,981	472.7	1.00	**66**	**66**	
		32,800	419.2	0.72	**58**	**58**	
		13,844	176.5	0.91	**56**	**56**	
20	Cervicovaginal				HPV^−^	72, 35, 52, 53, 54, 62, 81	No
21	Cervicovaginal				**HPV^−^**	**HPV^−^**	Yes
22	Cervicovaginal				HPV^−^	6, 53, 56, 62, 70	No
23	Cervicovaginal	135,138	1,711.5	0.73	**59**	**59**	Yes, partial
		110,107	1,392.2	0.99	**40**	**40**	
		95,442	1,193.2	0.99	87*		
		73,386	940.7	0.84	**67**	**67**	
		49,125	638.0	0.87	**73**	**73**	
		36,101	452.7	0.98	43*		
		29,721	375.9	0.97	**16**	**16**	
		13,891	171.4	0.73	**83**	**83**	
		9,380	119.9	0.99	**66**	**66**	
		3,091	39.5	0.99	68a*		
		2,121	27.0	0.85	**56**	**56**	
						45, 51, 61, 89	
24	Cervicovaginal	2503,879	31,916.9	0.99	**56**	**56**	Yes, partial
		2221,727	27,974.4	1.00	**52**	**52**	
		1054,024	13,048.1	0.99	**89**	**89**	
		598,662	7,506.7	0.90	43*		
		266,588	3,318.7	0.98	90*		
		213,130	2,692.1	0.95	**42**	**42**	
		50,171	628	0.66	**61**	**61**	
		40,905	523.9	0.99	**51**	**51**	
		31,221	390.3	0.98	87*		
						53, 68b	
25	Cervicovaginal				HPV^−^	66, 18, 31	No
26	Cervicovaginal	10277,696	129,998.7	1.00	**16**	**16**	Yes, partial
		428,009	5,411.7	0.97	**40**	**40**	
		184,266	2,310.5	0.86	43*		
		127,862	1,609.9	0.99	**52**	**52**	
		118,695	1,490.0	0.83	91*		
		90,888	1,148.0	0.99	**42**	**42**	
		3,722	46.0	0.66	**62**	**62**	
		2,610	33.3	0.96	**39**	**39**	
						72, 83	
27	Cervicovaginal				**HPV^−^**	26, 42, 58, 83, 84	No
28	Cervicovaginal	9,041	115.1	0.99	30*	35, 53	No
29	Cervicovaginal	23,813	295.1	0.76	114*	40, 54, 66, 84, 89	No
30	Cervicovaginal	2102,063	26,836.0	1.00	**39**	**39**	Yes
		1743,872	22,288.8	0.99	**66**	**66**	
		81,139	1,039.2	0.85	**51**	**51**	
		49,403	617.8	0.62	**6**	**6**	
		12,102	152.6	0.96	**11**	**11**	
31	Cervicovaginal				**HPV^−^**	**HPV^−^**	Yes
32	Plasmid	270,183	3,417.4	1.00	**16**	**16**	Yes

a Sample numbers 1 to 8 were multiplexed with control samples in pool 1, and samples 17 to 32 were multiplexed in pool 2. Sequence information is given only for those that passed the signal/noise cutoff for HPV type determination.

b Asterisks indicate HPV types not included in LA but detected by WGS. Boldface indicates types detected by both assays, while italics indicates types not included in LA assay.

c HPV concordance based on WGS and LA results.

d HPV64 has been reclassified as a subtype of HPV34. The only reference sequence available for HPV64 is an L1 fragment. On *post hoc* assessment, results for this sample are assigned to HPV64 based on reads mapped to the L1 fragment with good coverage and reads mapping to HPV34 with low genome coverage.

While some LA types were not detected by WGS in this small sample set (HPV26, -35, -53, -72, -81, and -84), other LA types were detected in some but not all samples (HPV18 and -66). In some instances, eWGS detected types but did not meet the criteria for number of reads or depth of coverage. For example, in sample 25, eWGS failed to call HPV66 and HPV18, which were detected by LA, but 1,478 reads mapped to HPV66 with 99% of the genome covered but failed because the average depth of coverage (18.9) was just below the cutoff of 20. HPV18 had a subthreshold of 234 reads mapped with 71% genome coverage. In sample 20, eWGS failed to call HPV72; although 1,412 reads mapped to HPV72 with 87% genome coverage, the average depth of coverage of 17.7 was below the cutoff of 20.

In sample 29, LA detected HPV84 but eWGS detected HPV114. HPV84 and HPV114 have 84% identity over the whole genome, suggesting the possibility of misclassification based on genomic fragments. To explore this, sample 29 reads were mapped with HPV84 as the only reference sequence. While no reads mapped to HPV84 under stringent conditions, under L0.5-S0.8 conditions 62,203 reads mapped to HPV84 with 94% of the genome covered. HPV53 was not identified in any of the 4 samples that were LA positive for this type; however, HPV53 sequences were found in one of four samples (number of reads, 1,438; average coverage, 18; genome coverage, 99%) if mapping was done under less stringent conditions (L0.5-S0.8).

LA includes probes for HPV64, which has been reclassified as a subtype of HPV34. For sample 17, 16,120 reads mapped to HPV34 with 16% coverage (compared to the whole genome), whereas 1,736 reads mapped to a 458-bp HPV64 L1 partial sequence (GenBank accession number AJ812226.1) with 85% coverage. As the full-length reference sequence for HPV64 is not available and HPV64 has been reclassified as a subtype of HPV34, the eWGS results were assigned to HPV64 based on *post hoc* assessment, giving partial concordance with LA results.

### Evaluation of custom HPV bait.

The performance of the HPV custom RNA bait pool for the 9 HPV types included as individual plasmids is shown in Table 3. One HPV6 bait sequence had to be removed due to homology with the human genome, resulting in 99.2% design coverage for that type; design coverage was 100% for the other 8 types. The mean coverage of mapped reads to reference sequences was 99.8% (range, 99.2 to 100%), and 99.2 to 100% of HPV reads mapped to the predicted type (mean, 99.60%). The mapped reads gave 95.9% uniform mean coverage (range, 92.6 to 96.3%), suggesting that the bait resulted in unbiased enrichment of the whole HPV genome. The eWGS method averaged 184,483-fold enrichment for HPV sequences in HPV-positive samples (range, 3,294 to 914,377).

**TABLE 3:** Performance of different RNA bait evaluation metrics based on WGS mapping results under L1-S1 from plasmid samples

Sample no.	HPV type^*a*^ (eWGS result)	Bait design coverage^*b*^ (%)	HPV genome coverage by mapped reads^*c*^ (%)	HPV reads mapped to predicted type^*d*^ (%)	Uniformity of coverage^*e*^ (%)
1	45	100	99.0	99.8	96.3
2	58	100	100	99.7	96.3
3	31	100	100	99.7	96.3
4	33	100	99.2	99.2	96.3
5	52	100	100	99.7	96.3
6	6	99.2	100	99.8	96.3
7	18	100	100	100	96.3
8	11	100	100	99.5	92.6
32	16	100	100	99.8	96.3
Mean		99.9	99.8	99.6	95.9

a HPV type determined by eWGS for the corresponding sample numbers (Table 2).

b Proportion of the HPV genome covered by the bait design criteria.

c Proportion of the HPV reference genome covered by the mapped reads.

d Proportion of reads that mapped to HPV predicted type compared to the total HPV reads.

e Uniformity of coverage across the genome was calculated as the percentage of bins with coverage within the average read depth ± 2 SD for all bins (see Materials and Methods for details).

## DISCUSSION

This is the first application of NGS for whole-genome identification of essentially all known HPV types (191 HPV types in the alpha, beta, and gamma genera) using RNA baits of the Agilent SureSelect target enrichment technology. The target enrichment method avoids the limitations of targeting only limited areas of the genome, minimizes the potential for PCR bias, and significantly increases the potential to increase the number of types identified in a single assay. A recent report using Roche NimbleGen DNA bait-based target enrichment largely focused on understanding the mechanistic signatures of integration of HPV types (87 types, 63% alpha) in cervical carcinomas (17), with limited data on NGS-based HPV type determination and concordance with a current HPV typing assay. In comparison, we mainly focused on the development of a universal HPV typing assay to detect all known HPV types in epidemiologic studies that increasingly examines a broad spectrum of HPV in alpha, beta, and gamma genera in both mucosal and cutaneous specimens for their disease association (5, 6, 11, 22). Toward this goal, we evaluated our method in terms of the performance of RNA baits for whole-genome identification, level of enrichment, and a number of read-mapping metrics for determination of HPV types under single and multiple infection.

The custom RNA bait library exhibited excellent performance in terms of the fraction of genome coverage, percentage of on-target reads mapped to predicted HPV types, uniformity of coverage for the types that we evaluated, and an observed average level of target enrichment (184,483) by a factor of nearly 5 log. Our results support the concept of specific capture and whole-genome sequencing of viruses from clinical samples through target enrichment technologies (18, 23, 24). Sequencing the target enriched libraries generated high-quality reads with more than 88% of bases having Q scores greater than 30. Mapping results revealed that this method could detect single and multiple infections of HPV along with HBG as an internal control for cellularity.

The mapping stringencies and threshold for the number of mapped reads required to avoid bleed-through of nonspecific HPV signals was established using 8 control samples (3 cell line controls at 100 ng, 3 cell line controls at 10 ng, human placental DNA at 100 ng, and water at 0 ng). The most stringent L1-S1 mapping conditions, along with a cutoff of ≥1,000 mapped reads, average coverage of ≥20, and fraction of genome covered of greater than 50%, allowed reliable sequence assignment. The control cell lines, Caski (∼500 HPV16 copies/cell), HeLa (∼50 HPV 18 copies/cell), and SiHa (1 to 2 HPV16 copies/cell), vary in copy numbers of HPV and were analyzed at two concentrations of input DNA (100 ng and 10 ng in 50 μl). In each case, the number of mapped reads under stringent conditions roughly correlated with copy number, and type assignment could be made with an input of 10 ng DNA. In agreement with previous reports using other methods (25, 26), the fraction of genome covered for HPV18 in HeLa cells was only 63%, since no reads mapped to a 2.6-kbp central region (nt 3100 to 5730), compatible with a deletion due to integration.

The bleed-through of reads from dominant sequences in adjacent clusters has been reported in multiplexed samples sequenced using the Illumina platform (27, 28). This might happen more frequently under relatively high seeding density for cluster generation and when one sequence dominates. These two conditions seemed to have taken place in this study. At the seeding density of 5.3 pM used in this study, pool 1 and pool 2 libraries generated cluster densities of 1,173,000 and 1,183,000, respectively, which is slightly higher than the recommended cluster density of 850,000 to 1,000,000. Additionally, over 31 million reads of HPV16 from Caski (100 ng) dominated sequences and corresponded to the HPV16 bleed-through found in other samples in the same pool. The HPV16 sequences in the placental DNA negative control in pool 1 had the unique 29 nucleotide substitutions identical to Caski HPV16 sequences (data not shown). It is unlikely that misidentification of barcode sequences contributed to bleed-through, since we restricted the analysis to reads with 0 mismatches in the index reads.

We applied the mapping stringency and cutoff determined with the control samples for determination of HPV types in 24 samples blinded to their HPV status. The HPV types determined by the eWGS were 100% concordant with LA in the 9 plasmid samples, but concordance was lower in the cervicovaginal samples (Table 2). Nine of 15 samples (60%) showed complete or partial concordance, and type-specific concordance for the 37 LA types was 95.83%. It could be anticipated that biologic samples from exfoliated cells would present greater challenges to HPV detection than purified plasmid DNA. These samples vary significantly in the amount of cellular and viral material. Given the relationship between viral copy number and number of reads noted in the plasmid and cell line results, it could be suspected that low copy numbers contribute to failure to detect types found by LA. Additionally, LA used 10 μl of extract regardless of the DNA concentration, while the eWGS method used 100 ng. Of the 4 samples that were LA positive but eWGS negative, three had 6 to 10 times (600 to 1,030 ng) more DNA input for LA. On the other hand, even with only 15 epidemiologic samples, eWGS detected 7 HPV types (HPV30, -43, -68a, -87, -90, -91, and -114) not targeted by LA. The ability of this eWGS method to detect HPV types not targeted by current assays indicates it could be useful in evaluation of HPV-negative cervical lesions (29) and in epidemiologic studies of HPV in geographic regions that may have uncommon types in circulation (30).

Some HPV types detected by LA were not detected by eWGS in this small sample set (HPV26, -35, -53, -72, -81, and -84). HPV84 was also reported as discordant between NGS and LA in earlier studies (10, 15). Further evaluation is needed to determine explanations for the failure to detect these types. Reduced mapping stringency did detect HPV53 and HPV84 in some instances and there could be unreported variants affecting mapping, and/or these types may be present at low copy numbers. We were able to confirm the specificity of RNA baits for HPV53 at the highest stringency of reference mapping by detecting HPV53 plasmid DNA (data not shown).

In conclusion, we developed and provided results from the initial evaluation of a non-PCR whole-genome sequence-based approach which is possibly close to a gold standard for broad-spectrum HPV genotype determination. The method relies on a custom-made RNA bait library that showed excellent performance in terms of genome coverage, percentage of reads mapped to predicted HPV type, uniformity of coverage, and the level of target enrichment. Further optimization and evaluation of this eWGS method for HPV detection is required in terms of cost per sample and throughput. Additional studies with samples including more types and optimization of sequencing conditions to minimize the bleed-through are needed. Studies evaluating the reproducibility and lower limits of detection are planned. Finally, it should be noted that despite enrichment with RNA bait by a factor of 5 log, 75% of the total reads (average) were human (off-target), with the exception of the sample with the highest viral load (100 ng Caski), in which 36% of total reads were off-target (data not shown). This suggests adjustment in the conditions for on-target enrichment could enhance results. Future laboratory and bioinformatics efforts are focused on determining the sensitivity and reproducibility of this HPV genotyping method, along with expanding its application for HPV detection and genotyping in samples from a variety of anatomical sites and development of a bioinformatics pipeline for automatic determination of HPV types and variants. This methodology is conceptually suitable for detecting both known and unknown HPV types through adjusting stringencies at the level of hybridization to RNA bait and reference mapping parameters. The current reference mapping stringency used to detect known HPV genotypes would likely miss unknown types.

## MATERIALS AND METHODS

### Samples.

DNA extracted from three cells lines (ATCC, Manassas, VA) known to include HPV16 (Caski, ∼500 copies/cell; SiHa, 1 to 2 copies/cell) and HPV18 (HeLa; ∼50 copies/cell) served as HPV-positive controls, and water and human placental DNA (Sigma-Aldrich Corporation, St. Louis, MO) were included as HPV-negative controls. A panel of coded samples was prepared so that testing and analysis could be performed in a blinded fashion. The blinded set included 9 residual samples from past WHO validation panels (whole HPV genome DNA plasmids diluted to 50,000 copies/sample in human placental DNA [100 ng/50 μl]) and 15 residual DNA extracts from anonymized cervical/vaginal samples previously typed using the Linear Array (LA; Roche Molecular Diagnostics, Indianapolis, IN). Two of the cervical/vaginal extracts were negative for the 37 genotypes targeted by LA, and the other 13 samples were selected to include multiple HPV genotypes (median, 5; range, 2 to 12). DNA was quantified by fluorescence-based PicoGreen assay (Molecular Probes, Inc., Eugene, OR), and volume was adjusted with a buffer (pH 8.0) composed of 10 mM Tris-HCl and 1 mM EDTA so that a final concentration of 100 ng/50 μl was used for library preparation. The exception was the water blank (0 ng DNA) and cell line samples that were also prepared at 10 ng DNA to test the effect of reduced DNA input on sequencing results. The final number of samples sequenced was 32: 8 control samples (3 cell line controls at 100 ng, 3 cell line controls at 10 ng, human placental DNA at 100 ng, and water at 0 ng) and 24 blinded samples (9 plasmid samples and 15 cervical/vaginal extracts [100 ng]).

### HPV whole-genome reference sequences.

We retrieved the whole-genome HPV reference sequences for all 188 HPV genotypes that were available in the PapillomaVirus Episteme (PaVE) database (http://pave.niaid.nih.gov/) as of September 2014 (31). We also retrieved whole-genome sequences for three HPV subtypes detected by LA (HPV55 [an HPV44 subtype], HPV82A/IS39, and HPV68b) from the National Center for Biotechnology (NCBI) database (http://www.ncbi.nlm.nih.gov/). The 191 HPV reference genomes included in this study (see Table S1 in the supplemental material) ranged in size from 7,095 to 8,104 bp and were distributed phylogenetically into 5 genera (67 alpha, 47 beta, 74 gamma, 2 mu, and 1 nu). The complete reference sequences were used for RNA bait design as well as for sequence mapping.

### Design and synthesis of RNA baits.

Agilent Technologies Inc. (Santa Clara, CA) designed a custom RNA bait library, using eArray software, comprised of 120-base RNAs complementary to one strand of the whole genomes of all 191 HPV genotypes at 2-fold coverage (60 bases overlapping). The resulting 23,941 RNA sequences comprised a bait library size of 1.442 Mbp. The bait library sequences were subjected to BLAST (Basic Local Alignment Search Tool) search against the NCBI human genome database, and 21 fragments were identified to have homology to human sequences. Removing these 21 fragments from RNA bait synthesis resulted in 100% coverage for 182 HPV genotypes and 99.17% to 99.25% coverage for 9 HPV genotypes (HPV6, -19, -34, -71, -82A/IS39, -142, -171, -172, and -175), for an overall design coverage of 99.96%. A BLAST search of the final synthesized bait sequences against the full NCBI database identified 15,669 unique hits, with 99.94% being specific to HPV sequences. The other 10 hits (0.064%) shared identity to chimpanzee, feline, and macaque papillomaviruses. As an internal control for the quality of samples, we included 12 RNA fragments covering nt 2041 to 3480 of the coding sequence of the human beta-globin (HBG) gene (1,439 bp; GenBank accession number GU324922.1). The custom biotinylated RNA bait library (HPV and HBG) was synthesized at Agilent Technologies. Upon request to the authors, the custom-designed ID may be shared with those who want to synthesize these probes through Agilent Technologies.

### Target enrichment library preparation and sequencing.

**FIG 4:**
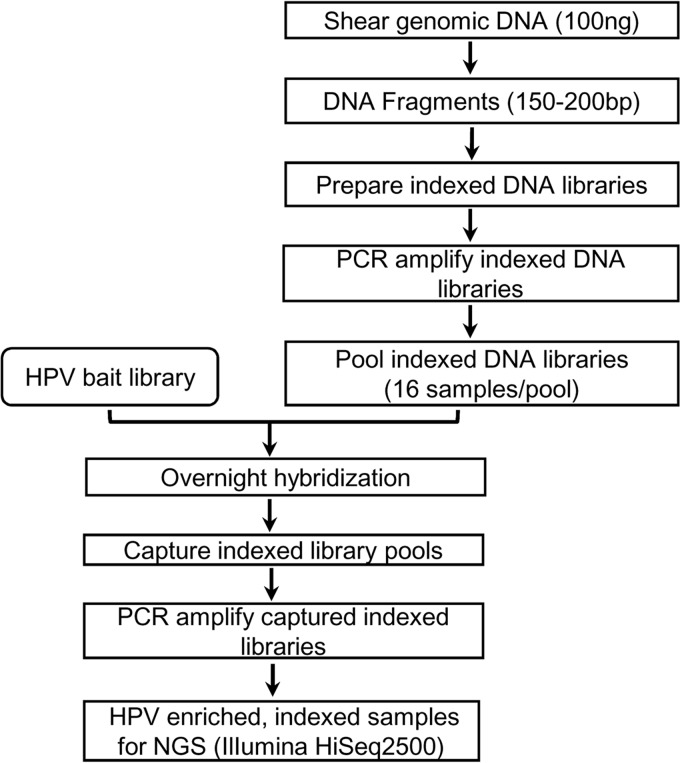
Laboratory workflow for HPV genotyping following RNA bait-based target enrichment and whole-genome sequencing.

The workflow for the preparation of the sequencing library followed Agilent Technologies' SureSelect^XT2^ target enrichment protocol (version D3), optimized for 100 ng DNA (Fig. 4). Briefly, DNA samples (50 μl in a 96-well microTUBE plate) were sheared using a Covaris LE220 focused ultrasonicator with SonoLab 7.3.2.4 software (Covaris, Inc., Woburn, MA). The conditions used for shearing (duty factor, 15%; peak incident power, 450; treatment time, 490 s) generated DNA fragments with a peak around 150 bp as determined by the high-sensitivity DNA kit and a Bioanalyzer 2100 (Agilent Technologies). DNA fragments were end repaired and 3′ A-tailed, and they underwent precapture indexing whereby each sample was barcoded with an index of 8 bp sequence. Indexed libraries were amplified by PCR with a limited number of cycles (8 cycles), followed by purification and assessment of the quality and quantity of each library by a Bioanalyzer 2100 (Agilent Technologies). Indexed libraries with unique barcodes were pooled (16 samples/pool) for overnight hybridization with custom RNA bait, followed by capture of hybridized fragments and 14 cycles of PCR to amplify indexed libraries. Following further purification, the quality and quantity of postcapture HPV enriched pooled libraries were assessed by a Bioanalyzer 2100 and quantitative PCR (qPCR) using a KAPA DNA library quantification kit (KAPA Biosystems, Wilmington, MA) and a LightCycler 480 (Roche Diagnostics, Indianapolis, IN). Pooled libraries were paired-end sequenced on a two-lane flow cell on Illumina HiSeq 2500 at the Centers for Disease Control and Prevention (CDC) Core Facility using TruSeq Rapid SBS kit HS (200 cycle) (Illumina, San Diego, CA) with Rapid run mode according to the following settings: read 1, 100 cycles; index (i7), 9 cycles; read 2, 100 cycles. For each DNA library, a seeding concentration of 5.33 pM was applied for cluster generation, with 5% PhiX virus genome library added as a sequencing control.

### Bioinformatics.

The raw sequence data were demultiplexed, and the adaptors and barcodes were removed using Illumina BCl2fastq V1.8.4. Reads with a base Q score were exported as fastq files for batch mapping to reference sequences using CLC Genomics Workbench 7.5 (CLC bio, Waltham, MA). The reference sequences of 191 HPV types/subtypes and HBG that were used for bait design were imported into CLC Genomics Workbench for mapping.

The analysis was directed to HPV detection and typing, so duplicate reads were not removed. We used reads with 0 mismatches in the index sequence for analysis in this report. Two parameters, read lengths (L0.5 or L1) and similarity scores (S0.8 or S1.0), were used to adjust mapping stringency. BAM (binary alignment/map) files were analyzed to generate mapping statistics by CLC Genomics Workbench. Three additional parameters were evaluated in the process of differentiating signal from noise: number of reads mapped to the HPV type-specific reference sequence, average depth of coverage for the mapped sequences, and fraction of HPV genome covered by mapped reads. HPV types in 8 control samples detected by eWGS with different mapping stringency and acceptance parameters were recorded prior to unblinding expected HPV results from 24 blinded samples. Subsequent matching of HPV genotype calls by WGS to expected results was used to evaluate performance of the assay and analysis.

RNA bait performance was evaluated based on results from single HPV plasmids: percentage of HPV reference genome covered by sequenced reads, percentage of reads that mapped to predicted types (compared to total HPV reads), and uniformity of coverage. To evaluate the uniformity, the reference sequences were divided into bins consisting of 300 bp, and the average mapping depth within each bin was calculated by Strand NGS software (http://www.strand-ngs.com/) using BAM files generated by CLC Genomics Workbench under L1-S1 mapping stringency. The mean and the standard deviation (SD) of the average mapping depths among the bins were calculated. The uniformity of coverage was calculated as the percentage of bins with coverage within the average read depth ± 2 SD for all bins. The target enrichment factor for a sample was calculated using the following formula: (total HPV mapped reads/total HPV and human mapped reads)/(HPV genome size/human diploid genome size) (32). HPV and human diploid genome sizes were considered to be 8 kbp and 6.6 Gbp, respectively, for this calculation.

## Supplementary Material

Supplemental material
