# Supplementary material for: Universal Human Papillomavirus Typing Assay: Whole-Genome Sequencing following Target Enrichment
Source: J Clin Microbiol. 2017 Feb 22;55(3):811–23. doi: 10.1128/JCM.02132-16 (PMC5328449; doi:10.1128/JCM.02132-16)
Supplement: Supplemental material [file supp_55_3_811__index.html]

Universal Human Papillomavirus Typing Assay: Whole-Genome Sequencing following Target Enrichment — Supplemental material 

# Universal Human Papillomavirus Typing Assay: Whole-Genome Sequencing following Target Enrichment

## Supplemental material

- Supplemental file 1 -

  Table S1 (HPV reference sequences used for design and synthesis of custom RNA bait library)

  XLSX, 22K
